# Zebrafish B cell acute lymphoblastic leukemia: new findings in an old model

**DOI:** 10.18632/oncotarget.27555

**Published:** 2020-04-14

**Authors:** Gilseung Park, Jessica Burroughs-Garcia, Clay A. Foster, Ameera Hasan, Chiara Borga, J. Kimble Frazer

**Affiliations:** ^1^Department of Cell Biology, University of Oklahoma Health Sciences Center, Oklahoma City, OK 73104, USA; ^2^Department of Pediatrics, Section of Pediatric Hematology-Oncology, University of Oklahoma Health Sciences Center, Oklahoma City, OK 73104, USA; ^3^Department of Microbiology and Immunology, University of North Carolina, Chapel Hill, NC 27599, USA; ^4^Department of Microbiology & Immunology, University of Oklahoma Health Sciences Center, Oklahoma City, OK 73104, USA; ^*^These authors contributed equally to this work

**Keywords:** acute lymphoblastic leukemia, ALL, zebrafish, lymphocyte, MYC

## Abstract

Acute lymphoblastic leukemia (ALL) is the most common pediatric, and ninth most common adult, cancer. ALL can develop in either B or T lymphocytes, but B-lineage ALL (B-ALL) exceeds T-ALL clinically. As for other cancers, animal models allow study of the molecular mechanisms driving ALL. Several zebrafish (*Danio rerio*) T-ALL models have been reported, but until recently, robust *D. rerio* B-ALL models were not described. Then, *D. rerio* B-ALL was discovered in two related zebrafish transgenic lines; both were already known to develop T-ALL. Here, we report new B-ALL findings in one of these models, fish expressing transgenic human *MYC* (*hMYC*). We describe B-ALL incidence in a large cohort of *hMYC* fish, and show B-ALL in two new lines where T-ALL does not interfere with B-ALL detection. We also demonstrate B-ALL responses to steroid and radiation treatments, which effect ALL remissions, but are usually followed by prompt relapses. Finally, we report gene expression in zebrafish B lymphocytes and B-ALL, in both bulk samples and single B- and T-ALL cells. Using these gene expression profiles, we compare differences between the two new *D. rerio* B-ALL models, which are both driven by transgenic mammalian MYC oncoproteins. Collectively, these new data expand the utility of this new vertebrate B-ALL model.

## INTRODUCTION

Acute lymphoblastic leukemia (ALL) and the related malignancy lymphoblastic lymphoma (LBL) dominate pediatric oncology, together representing over one third of all childhood cancer [1–3]. These diseases afflict even more adults in absolute terms, and are more often fatal in them [4, 5]. ALL and LBL can develop in lymphoblasts of either the B or T cell lineage; B-ALL exceeds T-ALL, but T-LBL is more prevalent than B-LBL. Despite treatments for these diseases improving considerably over the past several decades, relapsed ALL is actually the 4th most-frequent pediatric cancer diagnosis and second most lethal childhood malignancy, accounting for ~25% of all deaths [6–8].

Animal studies have been instrumental in investigating the genetics, genomics, drug sensitivities, and other features of ALL and LBL, and several mouse models for these diseases exist [9, 10]. Mammalian systems have advantages such as anatomic, physiologic, and genetic conservation, with mice being the main mammal utilized, but other organisms provide complementary advantages. Zebrafish (*Danio rerio*) offer flexible genetic tools, powerful live imaging, high fecundity, rapid development, shared oncogenic pathways, affordability, and other favorable traits that have allowed them to quickly gain traction as a cancer model [11–17].

Many groups have exploited these attributes to create zebrafish leukemia models, specifically for acute myeloid leukemia (AML) and T-ALL [18–37]. For T-ALL in particular, zebrafish models have been highly informative, advancing our understanding of T-ALL genetics, pro- and anti-oncogenic interactions between different genes and pathways, tumor heterogeneity, leukemia stem cells, and in screens for new therapeutics [28, 38–53]. However, despite the fact that zebrafish T-ALL models had proven to be fertile grounds for study, B-ALL modeling in *D. rerio* had not been fruitful, with only one low penetrance and long latency line reported [54]. This was curious because a zebrafish *recombination activating gene 2* (*rag2*) promoter—active in both immature T and B cells—was used to regulate most of these transgenic oncoproteins in the various T-ALL lines, yet *D. rerio* B-ALL had not been reported in them [55, 56]. Overall, since B-ALL is the more prevalent type in patients, the lack of B-lineage models was particularly unfortunate.

In 2018, the zebrafish ALL field advanced suddenly with reports of B-ALL in two closely-related transgenic lines where T-ALL was already known to occur [57, 58]. In one, a *rag2*:*mMyc* (murine *Myc*) transgene was used [29], with ALL purified as single clones by allo-transplantation. Two of 12 ALL analyzed by RNA sequencing (RNA-seq) exhibited gene expression consistent with B-ALL cells arrested at the pro-B cell stage [57]. In the other, a *rag2*:*hMYC* (human *MYC*) transgene was utilized [32], as well as a transgenic marker, *lck*:*eGFP* [59], differentially expressed by B and T cells. Analysis of over one hundred animals demonstrated that many develop B-ALL, others develop T-ALL (as previously known), and still other fish acquire both ALL types concommitantly [58]. A follow-up report by these groups further showed that despite high similarity between the *mMyc* and *hMYC* transgenes used, these B-ALL are actually quite different, occurring in distinct B cell lineages and with dissimilar expression patterns [60]. Here, we present new results in the *hMYC* model, including B- and T-ALL latency and penetrance data in a cohort of over 600 animals, *in vivo* glucocorticoid and radiation treatment of B-ALL, and expression profiles from single B- and T-ALL cells. We also present new analyses that compare *mMyc* vs. *hMYC* B-ALL to reveal key biologic pathways that distinguish them.

## MATERIALS AND METHODS

### Zebrafish care

Zebrafish care was provided as previously reported [58]. Animals were housed in an aquatic colony at 28.5°C on a 14:10 hour light:dark circadian cycle and experiments performed according to protocols approved by the University of Oklahoma Health Sciences Center IACUC (12-066 and 15-046). For all procedures, fish were anesthetized with 0.02% tricaine methanesulfonate (MS-222). *D. rerio* with the *cd79a*:*GFP* or *cd79b*:*GFP* transgenic markers [61] were bred to *rag2*:*hMYC* fish [32] to create the new transgenic lines reported herein.

### Fluorescent microscopy

Anesthetized 3–9 month old *hMYC; GFP* fish were screened for abnormal GFP patterns using a Nikon AZ100 fluorescent microscope. Low exposure (200 ms, 2.8× gain) and high exposure (1.5 s, 3.4× gain) settings were used to obtain images with Nikon DS-Qi1MC camera. Images were processed with Nikon NIS Elements Version 4.13 software.

### Fluorescence-Activated Cell Sorting (FACS) and flow cytometric analyses

As previously described [58], cells from whole fish were dissociated using a pestle, and then passed through 35 μm filters. GFP^hi^, GFP^lo^, and/or GFP^-^ cells were collected from the lymphoid and precursor gates using a BD-FACSJazz Instrument (Becton Dickinson, San Jose, CA, USA). Flow cytometric analyses were performed using FlowJo software (Ashland, OR, USA).

### B- and T-ALL incidence studies

Beginning at ~75 dpf, a cohort of 628 *rag2*:*hMYC*;*lck*:*GFP* zebrafish was monitored by ~weekly fluorescence microscopy to detect ALL. Animals that developed fluorescent cancers were euthanized and single cell suspensions were prepared as described previously [58]. Cells in the lymphoid and precursor gates were analyzed for GFP intensity using a Beckman-Coulter CytoFLEX™ to determine whether each ALL derived from T, B, or both lymphocyte lineages [58, 62, 63].

### *In vivo* dexamethasone and radiation treatments of *D. rerio* B-ALL

Zebrafish with B-ALL were treated by continuous immersion in 5 μg/ml dexamethasone (DXM) in normal fish water for 9 days, modified from our prior zebrafish T-ALL DXM protocol [43, 64]. Water and DXM were changed daily, with one or two fish housed in 500 ml. After completing treatment, animals were monitored by weekly fluorescent microscopy to detect relapse. Zebrafish with B-ALL were treated by γ-irradiation (IR) using a Cesium^137^ irradiator to deliver a total dose of 15 Gy divided in two fractions: 10 Gy on day 0 and a 5 Gy boost on day 5. Animals were imaged by fluorescent microscopy prior to treatment, 2 days post-treatment (day 7), and weekly-to-monthly thereafter to monitor for relapse.

### Nanostring™ gene expression analyses

FACS purification of normal lymphocyte and ALL samples from WT and *rag2*:*hMYC* fish, RNA extraction, and probe hybridization were performed as described previously [58], with gene identities and probe sequences available in the online supplemental material of that publication. Hybridization data were analyzed using nSolver 3.0 software (Nanostring nCounter Technologies, Seattle, WA, USA). Read counts were log-transformed and converted to z-scores for visualization purposes.

### Single-cell qRT-PCR analyses of *hMYC* B- and T-ALL

ALL cells from double-transgenic *hMYC* fish were isolated from peritoneal washings as described previously [63]. Individual B- and T-ALL cells were FAC-sorted into 96 well plates, and cells were lysed, RNA extracted, and cDNA synthesized according to the Fluidigm™ single-cell protocol. Twenty-cycle pre-amplification reactions were performed using gene-specific primers (listed in Supplementary Table 1.) according to manufacturer instructions. Unincorporated primers were digested with exonuclease I (NEB M0293L) and samples diluted 5-fold in DNA suspension buffer (TEKnova PN T0221). Single-cell pre-amplified cDNA were then quantified by qRT-PCR in a Fluidigm BioMark HD machine using 48.48 Dynamic Array Chips for Gene Expression following the manufacturer’s protocol. CT values were converted to Log2Ex values for visualization purposes using a limit of detection (LoD) of 28. Additional LoD limits were examined and showed similar results.

### RNA-seq and gene expression comparisons of *hMYC* and *mMyc* B-ALL

Paired-end RNA-seq reads were trimmed with BBDuk (v.38.22; retrieved from http://sourceforge.net/projects/bbmap) and aligned to the *D. rerio* GRCz11 genome using STAR (v.2.6.1b; default settings optimized for read-length) [65]. Picard’s MarkDuplicates tool (v.2.18.14; retrieved from http://broadinstitute.github.io/picard) was used to identify potential duplicates. Gene counts were generated with featureCounts (RSubread v.1.32.1) using *D. rerio* Ensembl annotation (release 92) [66, 67]. Ribosomal and mitochondrial RNA were excluded. Counts were processed and normalized within DESeq2 (v.1.22.1) [68], and pairwise differential expression testing was used to compare *hMYC* and *mMyc* B-ALL (adjusted *p*-value < 0.05, absolute fold-change > 1.5). Additional filtering steps were as described previously [60]. Putative human orthologues were mapped to corresponding *D. rerio* genes using BEAGLE (update 090718; retrieved from http://chgr.mgh.harvard.edu/genomesrus/index.php). In downstream analyses, a gene count threshold of 100 was used for each up-regulated group (minimum expression in at least 75% of in-group samples). RNA-seq heatmaps depict normalized counts generated via the variance-stabilizing transformation in DESeq2, unless otherwise described. Over-representation analysis was performed with clusterProfiler (v.3.10.0) [69] using putative human orthologues (FDR < 0.05) in the C2, C5, and C7 collections from MSigDB (v.7.0) [70]. The 10 highest-scoring pathways by FDR *q*-value for each group were chosen for visualization, with pathway names changed for brevity. Highly-redundant pathways were excluded manually, based on gene overlap. Original pathway names (order matching Figure 6B) and related data are listed in Supplementary Table 4.

## RESULTS AND DISCUSSION

### B-ALL is early onset and highly penetrant in *hMYC* zebrafish

**Figure 1:**
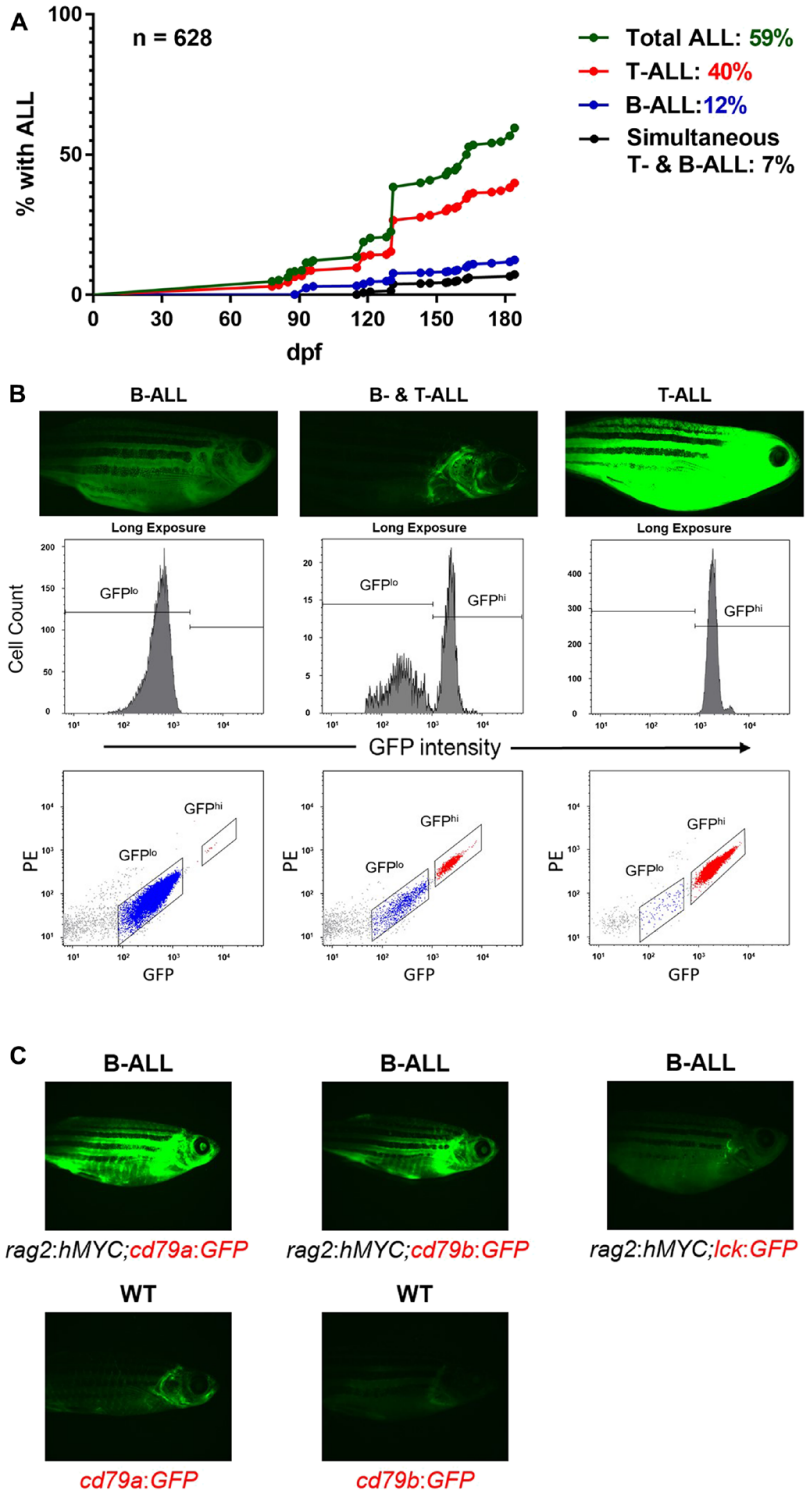
B-ALL in *hMYC* zebrafish. (**A**) Curves displaying B- and T-ALL incidence by 184 dpf, as determined by fluorescence microscopy. (**B**) Fluorescence microscopy images of fish with B-ALL (left), both B- and T-ALL (center), or T-ALL (right). Flow cytometry plots of ALL samples from these fish are shown beneath them. (**C**) B-ALL appearance in fish with *rag2*:*hMYC* and different transgenic markers: *cd79a*:*GFP* (left), *cd79b*:*GFP* (center), or *lck*:*GFP* (right). WT *cd79a*:*GFP* or *cd79b*:*GFP* fish without B-ALL are shown beneath for comparison. Images are representative of > 50 animals examined for each genotype.

We previously analyzed more than 50 unique *hMYC* B-ALL [58, 60], demonstrating these fish are a robust model, but latency and incidence rates for B- and T-ALL have not been reported in the *hMYC* line. To determine these, beginning at 3 months of age, we monitored fish by serial fluorescent microscopy to detect GFP^lo^ B-ALL and GFP^hi^ T-ALL, as we described previously (Figure 1A) [58]. Using the *lck*:*GFP* marker, GFP^lo^ B-ALL fluoresce dimly, making them difficult to discern by microscopy and more likely to require high disease burdens to be detected. In addition, brightly fluorescent T-ALL can obscure detection of B-ALL. To address these possibilities, we flow cytometrically tested ALL in order to definitively assign them to the correct group.

We surveyed over 600 fish until 184 days post fertilization (dpf) and found ~60% developed at least one form of ALL by that time (Figure 1A). Isolated T-ALL was most common (40%), with peak incidence between 4-5 months. B-ALL followed a similar time course, with 12% incidence by 184 dpf, and another 7% of animals developing both T- and B-ALL. To confirm these results, every ALL was analyzed flow cytometrically (Figure 1B). By 300 dpf, every surviving animal had developed at least one type of ALL (64% T-ALL, 23% B-ALL, 13% T- and B-ALL; data not shown). We conclude that ALL is highly penetrant in *hMYC* fish, with peak onset beginning shortly after 120 dpf.

To simplify detection of B-ALL by fluorescent microscopy and eliminate the need for flow cytometric confirmation, we created double-transgenic fish with *rag2*:*hMYC* and either the *cd79a*:*GFP* or *cd79b*:*GFP* marker (Figure 1C), which utilize promoters from B cell-specific proteins that are components of the surface immunoglobulin signaling complex [61]. In these lines, B-ALL fluoresce brightly, but T-ALL do not fluoresce. These models will be useful for B-ALL studies, particularly in investigations with potential therapeutic agents. The highly-fluorescent B-ALL of these lines can facilitate accurate and high-throughput determinations of both response rates and relapse kinetics, allowing precise regression measurements via fluorescence quantifications, and more sensitive detection of B-ALL relapses when disease burden still remains low.

### B-ALL respond to dexamethasone and radiation treatments

The glucocorticoids prednisone and dexamethasone (DXM) are the backbone of ALL therapy in patients, and γ-irradiation (IR) remains an effective adjunct that is also used, although it is reserved for specific cases due to toxicity [71, 72]. To determine whether B-ALL in zebrafish are similarly responsive to these modalities and establish protocols for future therapeutic studies in this model, we devised *in vivo* regimens for DXM and IR treatment of *D. rerio* with B-ALL.

**Figure 2:**
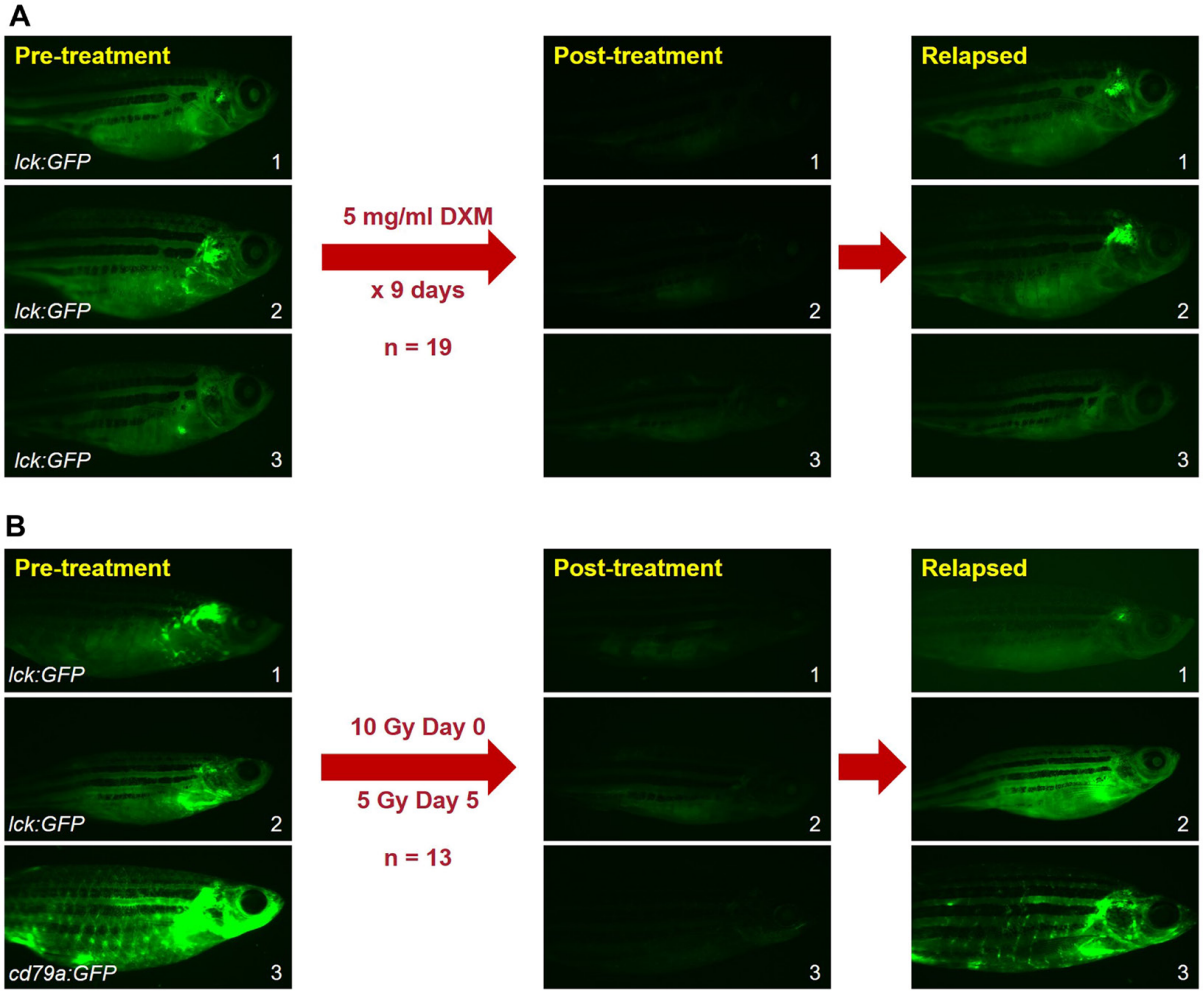
B-ALL regress with DXM or IR treatments. (**A**) B-ALL in *rag2*:*hMYC* fish treated with DXM for 9 days (*n* = 19). Post-treatment and relapsed images were obtained on days 9 and 28, respectively. Every (19/19) B-ALL regressed after DXM, but 11/19 (58%) B-ALL relapsed by day 28 (19 days after DXM withdrawal). (**B**) B-ALL in *rag2*:*hMYC* fish treated with IR (*n* = 13). Post-treatment and relapsed images were obtained on days 7 (i. e., 2 days after the 2nd IR dose) and 23, respectively. Every B-ALL (13/13) regressed after IR, but 100% recurred within 3 weeks of the 2nd IR dose. Upon relapse, flow cytometric testing (See Figure 1B) was used to verify that each relapse was GFP^lo^ B-ALL. Images of *rag2*:*hMYC*, *lck*:*GFP* fish (top 5 animals) have had brightness enhanced to facilitate visualization of dim B-ALL. Images of the *rag2*:*hMYC*, *cd79a*:*GFP* fish (lowest animal) are unmodified.

For DXM, fish were housed continuously in fish water containing 5 μg/ml DXM for 9 days, modified from our prior DXM studies in zebrafish T-ALL, and monitored by serial fluorescence microscopy [43, 64]. We treated nineteen animals, and every B-ALL showed robust regression, with most becoming undetectable by fluorescent microscopy by the end of treatment (Figure 2A). After completing DXM treatment, we then monitored fish for 9 weeks, with 58% (11/19) recurring by 4 weeks. We conclude that our DXM protocol effectively kills most B-ALL cells, as 100% of animals exhibited brisk responses, but in many cases, sufficient B-ALL cells persist to re-grow the cancer quickly. Based on this, we believe this model can be utilized to investigate the genetic and molecular features of steroid-resistant ALL, a significant clinical problem in ALL patients [73].

IR is toxic in patients, and we also observed this in zebrafish with B-ALL. We initially administered IR treatments of 20 Gray (Gy) as single doses, but many animals died, despite the fact that this dose is well-tolerated by wild-type (WT) fish [59]. To diminish IR toxicity, presumably caused by overwhelming tumor lysis [74], we divided IR into two fractions, giving 10 Gy on day 0 and an additional 5 Gy on day 5. This was better tolerated, and following the second fraction, B-ALL were undetectable by fluorescence microscopy (Figure 2B). However, as seen in many DXM-treated B-ALL, all B-ALL relapsed post-IR, and recurrences were even more rapid (~3 weeks). From these results, we infer that IR (like DXM) kills most B-ALL cells, but surviving cells quickly re-populate the tumor. In addition, the rapid re-growth of B-ALL after IR suggests either more cells persist following IR, or that B-ALL cells that survive IR adopt more aggressive phenotypes. Distinguishing between these possibilities and unravelling the underlying mechanisms responsible will be informative to the clinical scenarios these experiments represent.

### Defining B-ALL and B cell gene expression patterns

We discovered many *D. rerio* B lymphocytes and B-ALL express low levels of *lck* [58], which fostered new studies not previously done in zebrafish, such as FACS isolation of B-lineage cells (i. e., GFP^lo^ cells from *lck*:*GFP* fish) and profiling their gene expression [58, 63]. These findings translate to humans because immature human B cells and many patients’ B-ALL also express low levels of *LCK* [58, 75, 76]. It is currently not known if these newly-described *lck*^lo^/*LCK*^lo^ B cells represent one, or many, B cell population(s).

**Figure 3:**
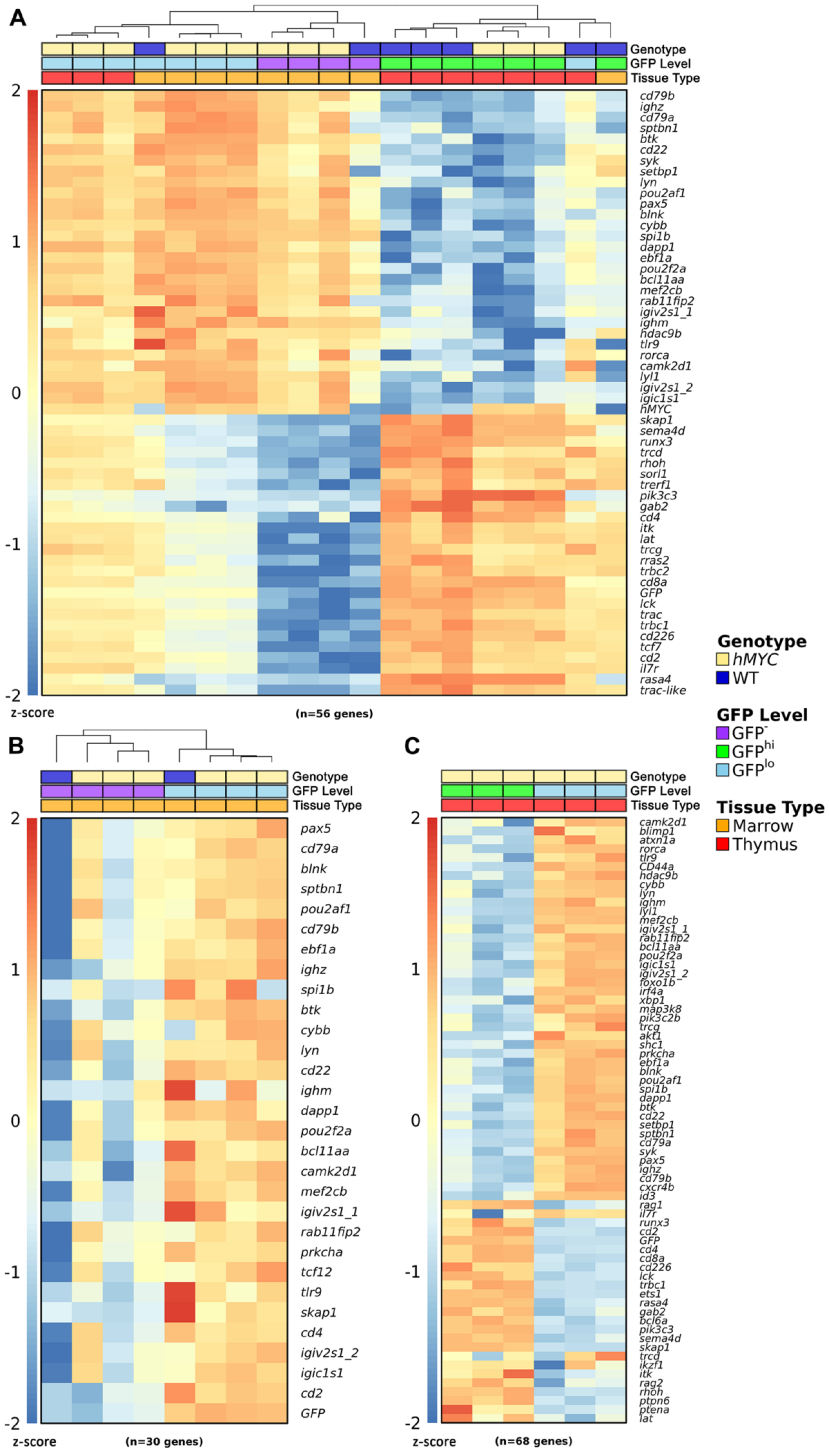
Gene expression in zebrafish lymphocytes. (**A**) Nanostring™ expression data in GFP^-^, GFP^lo^, and GFP^hi^ lymphocytes from WT or *rag2*:*hMYC* fish bearing the *lck*:*GFP* marker transgene. *hMYC* GFP^lo^ cells from thymus and marrow express B cell genes, as do WT GFP^lo^ marrow cells. *hMYC* and WT GFP^-^ marrow cells also express B cell transcripts. WT and *hMYC* GFP^hi^ thymocytes express T cells genes, as do WT GFP^hi^ marrow cells. WT GFP^lo^ thymocyte fractions contain a mixture of B and T cells (as do *hMYC* GFP^lo^ thymocytes and WT GFP^lo^ marrow cells), due to the difficulty of obtaining pure GFP^lo^ populations from these tissues [58, 63]. Samples are clustered hierarchically. (**B**) Nanostring™ expression data for B cell genes in marrow lymphocytes. WT and *hMYC* GFP^lo^ marrow cells show the strongest B cell signature, but B cell hyperplasia in *hMYC* fish increases the B cell signature of GFP^-^ *hMYC* marrow cells compared to WT GFP^-^ marrow cells, which show little-to-no B cell gene expression. Samples are clustered hierarchically. (**C**) Nanostring™ expression data in *hMYC* thymocytes. *hMYC* GFP^lo^ thymocytes express B, but not T, cell genes; *hMYC* GFP^hi^ thymocytes show the opposite pattern. In panels (A–C), triplicates [*hMYC*/GFP^lo^/thymus (tan/lt. blue/red), *hMYC*/GFP^lo^/marrow (tan/lt. blue/orange), *hMYC*/GFP^-^/marrow (tan/purple/orange), WT/GFP^hi^/thymus (dk. blue/green/red), and *hMYC*/GFP^hi^/thymus (tan/green/red)] represent individual biologic replicates, where each column depicts results from RNA of cells pooled from 10 fish. For each singleton sample [WT/GFP^lo^/marrow (dk. blue/lt. blue/orange), WT/GFP^-^/marrow (dk. blue/purple/orange), WT/GFP^lo^/thymus (dk. blue/lt. blue/red), and WT/GFP^hi^/marrow (dk. blue/green/orange)], cells were pooled from 30 fish of the indicated genotype for RNA extraction. In panels A–C, read counts were log-transformed and converted to z-scores for visualization purposes. Scale bars are shown at the left of each heatmap.

To begin to address this, we assessed gene expression in lymphocytes of fish carrying only the *lck*:*GFP* marker transgene (henceforth referred to as WT) and double-transgenic *rag2*:*hMYC*, *lck*:*GFP* fish (henceforth, *hMYC*). We FACS-purified lymphoid gate cells from both marrow and thymus, and further divided these into GFP-negative (GFP^-^), GFP^lo^, and GFP^hi^ fractions [58, 62]. We then used Nanostring™, a multiplexed probe-based hybridization technique, to quantify mRNAs expressed by each population [77, 78]. To categorize distinct cell identities, we selected zebrafish gene homologues whose expression distinguishes mammalian B, T, and other leukocytes for these experiments [58, 79]. We found that GFP^hi^ cells from both thymus and marrow in both genotypes (WT, *hMYC*) express T-lineage transcripts such as *cd2*, *cd4*, *cd8a*, *itk*, *lat*, and T cell receptor (TCR) mRNA (*trcd*, *trcg*, *trbc2*, *trac*, *trbc1*; Figure 3A). RNA-seq studies by other groups indicate some *D. rerio* natural killer (NK) and myeloid cells also express *lck* [49, 80], but transcripts indicative of these cells were not seen, suggesting they are minor populations in marrow and thymus. In contrast, B cell gene expression (*cd79a*, *cd79b*, *ighz*, *btk*, *cd22*, *syk*, *lyn*, *pax5*, *blnk*, *ighm*, etc.) was prominent in GFP^lo^ and GFP^-^ fractions from both organs (Figure 3A), particularly in *hMYC* GFP^lo^ cells of both marrow (Figure 3B) and thymus (Figure 3C), where B-lineage genes were the dominant signature. We draw two inferences from these data: (1) *lck*^-^ and *lck*^lo^ lymphocytes both contain B cells, but the *lck*^lo^ population has greater B cell enrichment and (2) *lck*^lo^ B cells are more abundant in *hMYC* fish, as B-lineage genes were more dominant in *hMYC* GFP^lo^ signatures than in those of WT animals.

These results support our conclusions, but using bulk gene expression patterns to compare populations that contain multiple cell types is challenging. Alternatively, single cell analyses can unambiguously define cell identities and precisely quantify different cell types. Such approaches can also reveal heterogeneity in seemingly-uniform populations, which is more powerful than binary categorizations like simple lineage assignments. Recognizing differences between cells of the same lineage, or cells of the same cancer, also has functional relevance, since distinct gene expression patterns correspond to different cellular phenotypes. Lymphocytes are diverse and complex populations, composed of not only B, T, and NK cells, but also innate lymphoid cells (ILC) [81]. Moreover, each of these cell types have multiple additional subtypes, such as the distinct IgM and IgZ B cell lineages in teleost fish [61, 82–84] or the various T lymphocyte subtypes [e. g., cytotoxic (CD8^+^ CTL), helper (CD4^+^ T_H_), T_H_17, and regulatory (T_reg_) subclasses] [85–87].

**Figure 4:**
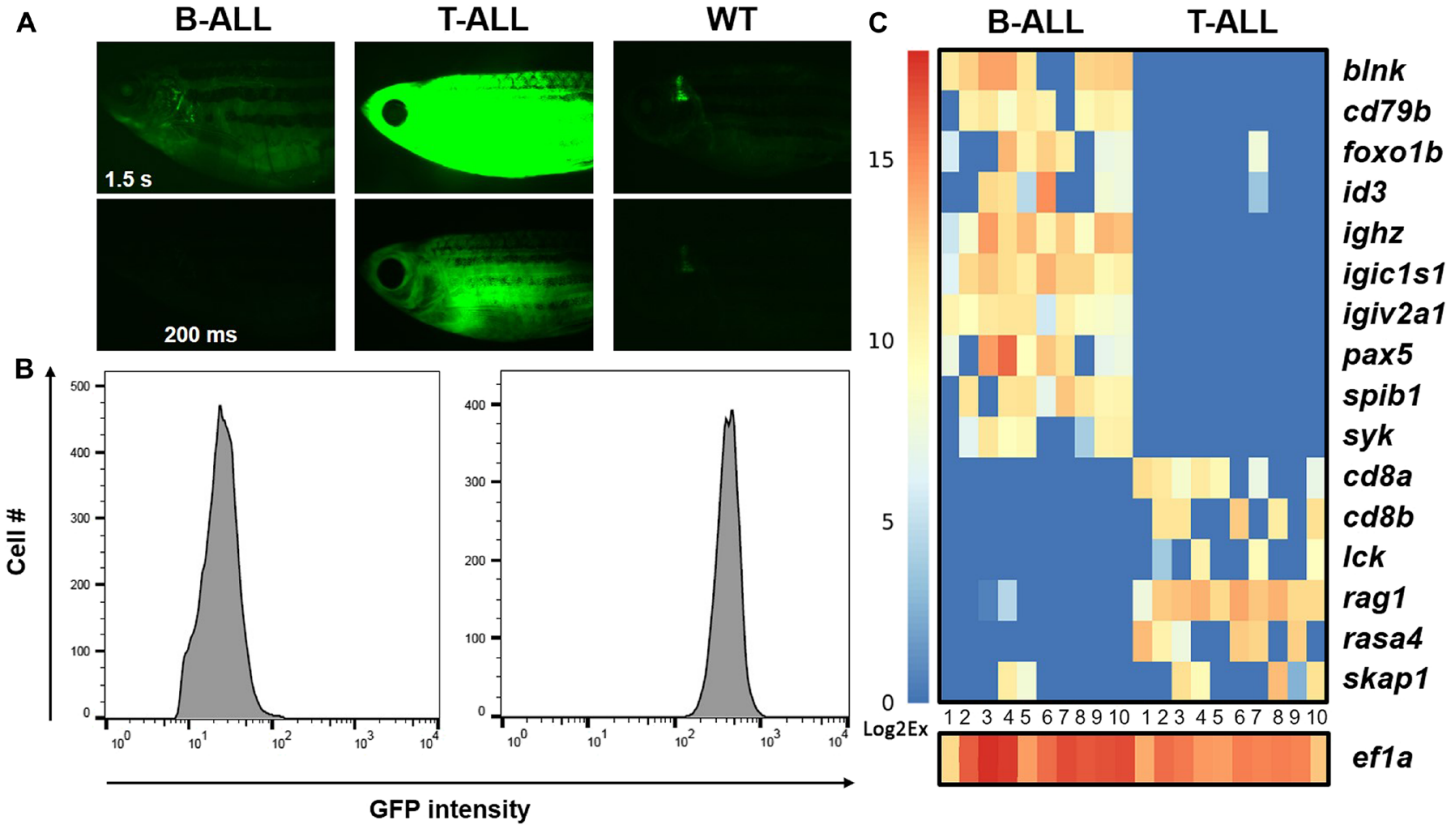
Gene expression in individual *hMYC* B- and T-ALL cells. (**A**) *rag2*:*hMYC*;*lck: GFP* fish with dim (left) and bright (center) cancers. B-ALL is only visible with 1.5 s exposure (upper left); T-ALL is visible with either 1.5 s (upper middle) or 200 ms (lower middle) exposures. Control *lck*:*GFP* fish images are shown at right, where only the thymus is visible. (**B**) Flow cytometry plots of the same ALL samples demonstrate GFP^lo^ B-ALL and GFP^hi^ T-ALL specimens. (**C**) Gene expression in single B- and T-ALL cells of the same cancers (*n* = 10 cells for each), as determined by Fluidigm™ Biomark qRT-PCR. B-ALL cells express B cell, but not T cell, genes; T-ALL show the opposite pattern. Expression of *eef1a1l1* (homologue of human *eukaryotic translation elongation factor 1 alpha 1*, aka *EF1A*) was used as a threshold control for the presence of RNA in each well. CT values were converted to Log2Ex for visualization (scale bar at left).

Diversity is not unique to normal lymphocytes; lymphoid cancers also contain heterogeneous populations, as previously shown in *D. rerio mMyc*-driven T-ALL [15, 40, 45, 48]. To assess tumor heterogeneity in *hMYC*-induced ALL and begin to define its extent, we analyzed mRNA expression in single cells from B- or T-ALL from *hMYC* fish (Figure 4). Animals exhibited either dim or bright cancers by fluorescent microscopy (Figure 4A) and by FACS analysis (Figure 4B). Using Fluidigm Biomark™ multiplex quantitative reverse transcriptase polymerase chain reaction (PCR primers are listed in Supplementary Table 1), we analyzed the expression of 17 transcripts: 10 B-lineage, 6 T-lineage, and as a mRNA threshold control, *eukaryotic translation elongation factor 1 alpha 1, like 1* (*ef1a*) in 10 B- and 10 T-ALL individual cells (Figure 4C). All 20 B- and T-ALL cells profiled in accordance with their expected lineage, but expression differences of specific genes in single cells (e. g., *pax5*, *id3*, etc.) were evident. In addition, select genes (e. g., *foxo1b*, *skap1*) occasionally were mis-expressed by cells of the ‘wrong’ lineage. The significance of these findings remain to be determined, as malignant cells express aberrant markers on occasion [88], but biphenotypic ALL was demonstrated in *mMyc* fish, so what has previously been termed gene ‘mis-expression’ may actually represent an intriguing finding [57, 60]. Future single-cell studies hold promise as a means to address these and other unanswered questions about the cellular heterogeneity amongst lymphocytes, and within individual cancers, so as to define the precise gene expression differences that underlie their differing cellular phenotypes *in vivo*.

### Distinct types of B-ALL driven by *hMYC* and *mMyc*

**Figure 5:**
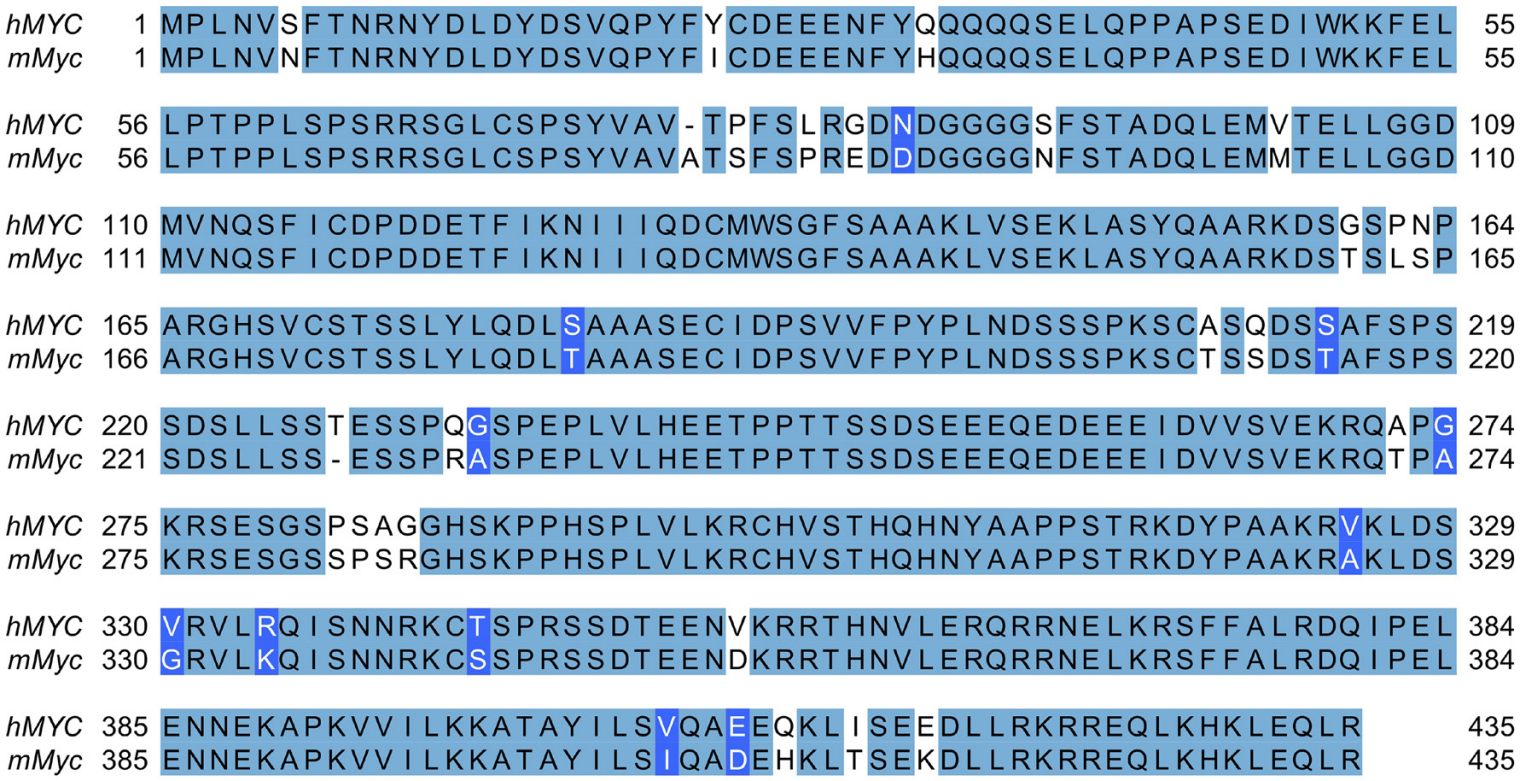
Alignment of human and murine MYC proteins. Transgenic human and mouse MYC are 435 and 439 amino acids, respectively (last four *mMyc* residues not shown). Proteins show > 91% identity (399 residues, shaded light blue) and > 94% similarity (410 residues; 11 additional similar residues shaded darker blue).

MYC acts oncogenically in many ALL cases, and MYC is hyperactive in several other forms of cancer also [89], so it is not surprising that *hMYC* and *mMyc* can induce ALL in several types of lymphocytes [60]. However, the human and mouse MYC oncoproteins in these transgenic fish are highly conserved (435 amino acids, ~92% identical, ~94% similar; Figure 5), and both lines regulate MYC using the same zebrafish *rag2* promoter, so it was surprising to discover that B-ALL in *hMYC* and *mMyc* fish are quite different. Specifically, *hMYC* B-ALL arise in *ighz*-lineage precursor B cells, whereas *mMyc* B-ALL occur in pro-B cells of the *ighm* lineage [60]. Unlike mammals which lack IgZ, expression of the zebrafish IgZ isotype does not occur by class switching. Instead, because of the configuration of the *D. rerio* immunoglobulin heavy chain (IgH) locus, during VDJ recombination zebrafish B cells must delete *ighz* sequences in order to express IgM [82]. Thus, when B cells commit to the IgM lineage, they lose their ability to express IgZ. As such, IgZ B cells, which are believed to primarily mediate mucosal immunity, can be viewed as an earlier stage in zebrafish B cell development that precedes IgM lineage commitment.

**Figure 6:**
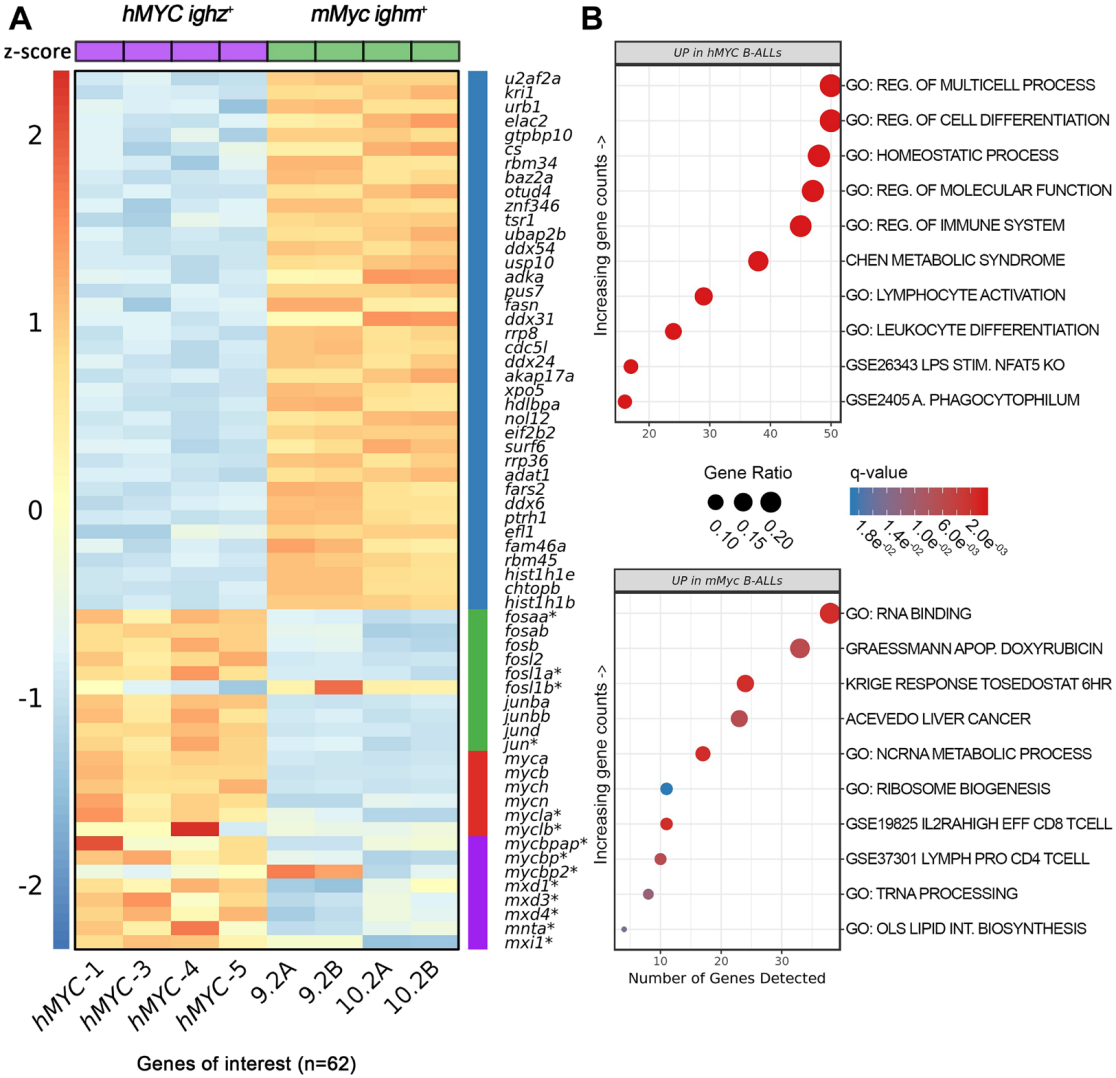
Differentially-expressed genes in *hMYC* versus *mMyc* B-ALL. (**A**) Heatmap depicting 62 differentially-expressed mRNA, including genes of the Gene Ontology (GO) RNA binding pathway (blue bar), *D. rerio fos*/*jun*-family members (green bar), endogenous *myc*-family members (red bar), and other *myc*-related proteins, including several *max* heterodimeric partners (purple bar). Samples *hMYC-1*, *-3*, *-4*, and *-5* are four distinct *hMYC* B-ALL; 9.2A/B and 10.2A/B pairs are RNA-seq technical replicates of two *mMyc* B-ALL [57, 60]. Genes not meeting differential expression testing thresholds or other filtering criteria are marked by asterisks (FDR < 0.05, absolute fold-change ≥ 1.5). Read counts are shown as z-scores (scale bar at left). (**B**) Top ten biologic pathways up-regulated in *hMYC* (top) or *mMyc* B-ALL (bottom; FDR < 0.05). Pathways are ordered according to the number of genes detected in the gene set (x-axis) and colored based on FDR *q*-value. Data point sizes correspond to the percentage of genes over-expressed in each pathway.

We used RNA-seq to profile gene expression in *hMYC ighz*^+^ and *mMyc ighm*^+^ B-ALL (Figure 6) [60]. We compared 4 *hMYC* B-ALL to duplicate transcriptomes from the only 2 *mMyc* B-ALL thus far reported [57]. As noted, this revealed *hMYC* B-ALL expressed *ighz* constant regions while *mMyc* B-ALL expressed *ighm* constant region mRNA [60], plus additional differences. Overall, > 400 genes displayed statistically-significant differential expression (Figure 6A and Supplementary Tables 2 and 3), including several pathways of interest. Curiously, in *hMYC* B-ALL much higher expression levels were seen for 9 of 10 *fos* and *jun* family members in the zebrafish genome (green bar in Figure 6A) with the lone exception *fosl1b*, which was higher in *mMyc* B-ALL. Also intriguing was the finding that all 6 endogenous *D. rerio* MYC family members (*myca*, *mycb*, *mycn*, *mych*, *mycla*, and *myclb*; red bar in Figure 6A) were more highly expressed in *hMYC*-driven B-ALL, as were multiple MYC-binding proteins and several MAX proteins, which heterodimerize with MYC (purple bar in Figure 6A).

B-ALL from the *mMyc* model showed dramatic up-regulation of many genes belonging to the Gene Ontology (GO) RNA binding pathway (blue bar in Figure 6A and top entry of Figure 6B lower panel). Several other genes also showed higher expression in *mMyc* B-ALL (Supplementary Table 3), including *D. rerio* homo/orthologues of mechanistic target of rapamycin kinase (*MTOR*) and the MTOR complex 1 member *RPTOR*, multiple cyclin-dependent kinases and related cell cycle regulators (*CDK5*, *CDK14*, *CDC5L*, *CACUL1*, *TNK2*), and the non-receptor tyrosine phosphatases *PTPN13* and *PTPN21.* Together, these results suggest cell growth and division may be regulated differently in *mMyc* vs. *hMYC* B-ALL. Other notable orthologues up-regulated in *mMyc* B-ALL included the *MYB*-like gene *MYSM1*, two genes with jumonji lysine demethylase domains (*JMJD8*, *JARID2*), and the transcription factor *NFAT5*.

We postulate these and other differences may explain the apparently disparate oncogenic mechanisms employed by *hMYC* and *mMyc* in the B lymphoblasts of these closely-related lines. Pathway analysis of differentially-regulated genes predicted differing activation of several biologic pathways (e.g., cell differentiation, immune system process, lymphocyte activation, RNA binding, etc.; Figure 6B panels and Supplementary Table 4). These markedly different pathway signatures further demonstrate that human and murine MYC are far from synonymous in terms of their oncogenic effects upon zebrafish B lymphoblasts.

## CONCLUSIONS

Recent discoveries of B-ALL in *D. rerio* lines previously-known to develop T-ALL were unexpected, but in retrospect, are not surprising. Both B and T lymphoblasts express *rag2*, so it is predictable that the transgenic promoters used in *hMYC* and *mMyc* fish would be active in both lymphocyte lineages. Likewise, MYC is known to be potently oncogenic in multiple types of B cell cancer, so the fact that mammalian MYC proteins can induce zebrafish B-ALL is no more surprising than their already well-documented activities in promoting zebrafish T-ALL. Rather, what is somewhat surprising is that over a decade passed between the first description of *rag2*:*mMyc* fish and the recognition that B-ALL occurred in them. This may reflect that B-ALL is less prevalent than T-ALL, but our incidence data in the related *rag2*:*hMYC* line (Figure 1A) demonstrate that B-ALL is still quite common—at least in *hMYC* fish. Irrespective of this, now that B-ALL are described, recognizing them is straightforward, either by differential GFP expression in the *lck*:*GFP* background, or with other transgenic markers like *cd79a*:*GFP* or *cd79b*:*GFP* (Figure 1C). In the case of these latter two lines, T-ALL will of course still develop, but go undetected. In view of this, it is worth noting that other forms of ALL are perhaps yet to be discovered in *mMyc* and *hMYC* fish, as alluded to by the discovery of a biphenotypic ALL of mixed B/T-lineage in the aforementioned *mMyc* study [57]. Going forward, continued efforts to discern the molecular mechanisms by which MYC mediates oncogenic effects in different lymphocyte lineages promise to yield even more insights into how this potent oncogene can drive cancer in zebrafish, and in humans.

## SUPPLEMENTARY MATERIALS








