# Supplementary material for: Zebrafish B cell acute lymphoblastic leukemia: new findings in an old model
Source: Oncotarget. 2020 Apr 14;11(15):1292–305. doi: 10.18632/oncotarget.27555 (PMC7170496; doi:10.18632/oncotarget.27555)
Supplement: Supplementary file 1 [file oncotarget-11-1292-s001.pdf]

Zebrafish B cell acute lymphoblastic leukemia: new findings in an old model

SUPPLEMENTARY MATERIALS

Supplementary Table 1: Primer sequences for outer preamplification and inner qPCR reactions

96 primers on IFC	Purpose	Outer Forward Primer Sequence	Outer Reverse Primer Sequence	Inner Forward Primer Sequence	Inner Reverse Primer Sequence
blnk	SC qRT PCR	CAACCCAGCAAACAACCCAG	GACTCCTCTGTTCACCACCG	CGAGGACGACTACATCGAGC	GTCAGTGTTCTCTCCGTGGG
cd79b	SC qRT PCR	TTGGCGTTAAGACAGGTCGG	GTCGAACCAGAGGAACGACA	TACTGTGTGCCGTCGAATCC	GTGCCACTGTCCTCAGTCTC
foxo1b	SC qRT PCR	GACCCGGATTTTGAGCCTCT	GTTGTCCTGGCAGTGGAAGT	AAACCGGAGCAAGGAATCGT	GGCTTCTCGTCAGGGTAGTC
id3	SC qRT PCR	AAGCTGCTATGAAGCGGTGT	AATCTCCACTTGGCTCACGG	CTGCAAGAGTCCTTCCGAGG	TGGCTCACGGACTTGTTCTG
ighz	SC qRT PCR	CCACCCGGGACACGCTTAAT	TGGAGCCCTGTTGTTGGATG	CAGGAGACACTGTGAGGAG	TAGCAGGAGTGTGTGTGTGC
igic1s1	SC qRT PCR	GAGCAGCAGTGGATGGAGAG	AGTGTGTGTGCTGAATGCAG	CAGGAGACACTGTGAGGAG	TAGCAGGAGTGTGTGTGTGC
igiv2a1	SC qRT PCR	CACTGTGATGGTGCTTTGGC	TCGCCCACTTTCCAGCTTAG	GATGAGCTGCAGAAGGGTCA	GCGTCCAGTCAGAAGGGAAT
pax5	SC qRT PCR	CAGCACAACACTCCCAGGAT	AATAGTCCCGATGACGCTGC	AAGGCAGTTACTCCACACCC	ACCGTACTCCTGCTGAAACAC
spib1	SC qRT PCR	CTCACAACGTCCAGCCATCT	TTGCGATTGCCCTTCTGGAT	ATCCCAGCAGTCGTAGTCCT	TCTCGATCCACCCACCAGAT
syk	SC qRT PCR	TGAACGACACCTACGCCATC	CCGTTGACCCGTGAGGTATT	AGCTGGAGAAACTCATCGCC	GCAAACGCAGCTCTGAATCC
cd8a	SC qRT PCR	TGCAAAAAGGACAGACAGCG	AAAGTCCACAACCTCCGACC	ACTCTTCTTCGGAGAGGTGAC	ACAGGCTTCAGTGTTGTTTGAA
cd8b	SC qRT PCR	AAGGTGTCCTACAACCTGCG	GCCAAAAGCAGAAGAGCACC	AGCGAGAAGATGCTGGCTTT	CGGCTGAAGTGTTGGAGGAT
lck	SC qRT PCR	AGATTGCTGACTTCGGCCTG	GTAGCCCCTCTCGAGGTTTG	GGCACCAGAGGCCATAAACT	CTCTGGGTTTGTCATTCCTGG
rag1	SC qRT PCR	CCAGGTGAAGACATTTGCCG	ATTACGCAGAGTGTGCAGGG	AGCAATGATGCAAGGCAGAG	TGTGCAGGGGCTGGAATATC
rasa4	SC qRT PCR	GATCCGTGCGGTAGAGAAGG	AGATGAGCTGAGCCTCCAGA	TGGAGGAGAAGTGTTTCGGC	TCTGCTTTGTAGATGCCGGG
skap1	SC qRT PCR	AAAGACCAAGCACTGCCTCA	CCTCTCTGGAAGGCCAACTC	GCAGTGAGAGAACTGGGTCC	CGGTCCCTCAGCTTCACAAT
ef1a	SC qRT PCR	AGCGTGGTATCACCATTGACA	TTCACTCCCAGGGTGAAAGC	GAGACCAGCAAATACTACGTC	GGAGATACCAGCCTCAAACTC

Supplementary Table 2: Differentially-regulated genes in Figure 6A. See Supplementary Table 2

Supplementary Table 3: Differentially-expressed genes in *hMYC* vs. *mMyc* B-ALL. See Supplementary Table 3

Supplementary Table 4: Pathways differing between *hMYC* vs. *mMyc* B-ALL. See Supplementary Table 4
